# Supplementary material for: The genome sequence of the Grouse Louse Fly, Ornithomya chloropus (Bergroth, 1901)
Source: Wellcome Open Res. 2025 Apr 11;10:191. [Version 1] doi: 10.12688/wellcomeopenres.23962.1 (PMC12284471; doi:10.12688/wellcomeopenres.23962.1)
Supplement: Peer review history

# Reviewer report: Reviewer response for version 1

**DOI:** 10.21956/wellcomeopenres.26438.r126114

**Published:** 2025-07-22

**Authors:** Hans-Peter Fuehrer

**Affiliations:** 1 University of Veterinary Medicine, Vienna, Austria

The authors describe the genome sequence of a grouse louse fly Ornithomya chloropus. The manuscript is of very good quality and just  some minor modifications are recommended.

 Background: Please change fly to dipteran or ked.

 The English name for louse flies is keds.

 Initial identification by barcoding. Where there sequences to compare with?

Are sufficient details of methods and materials provided to allow replication by others?

Yes

Is the rationale for creating the dataset(s) clearly described?

Yes

Are the datasets clearly presented in a useable and accessible format?

Yes

Are the protocols appropriate and is the work technically sound?

Yes

Reviewer Expertise:

Medical and veterinary entomology

I confirm that I have read this submission and believe that I have an appropriate level of expertise to confirm that it is of an acceptable scientific standard.

# Reviewer report: Reviewer response for version 1

**DOI:** 10.21956/wellcomeopenres.26438.r126113

**Published:** 2025-07-12

**Authors:** Komal Kumar Bollepogu Raja

**Affiliations:** 1 Baylor College of Medicine, Houston, Texas, USA

Wawman D.C. and Insley H. present the genome sequence of the species *Ornithimya chloropus*. The genome sequence length is 193.17 megabases. The authors have sufficiently provided the ecological and the morphological features of this species in the background. The experimental methods and the bioinformatics tools used in the manuscript are standard and can be reproducible. The genome assembly quality metrics look great with 96% BUSCO and 99% combined k-mer completeness. Further, 180x genome coverage with 99.5% mapped to chromosomes is great. Overall, the manuscript is well written and the data is a good genomic resource.

Are sufficient details of methods and materials provided to allow replication by others?

Yes

Is the rationale for creating the dataset(s) clearly described?

Yes

Are the datasets clearly presented in a useable and accessible format?

Yes

Are the protocols appropriate and is the work technically sound?

Yes

Reviewer Expertise:

Genomics, single cell genomics, bioinformatics and neurobiology

I confirm that I have read this submission and believe that I have an appropriate level of expertise to confirm that it is of an acceptable scientific standard.

# Reviewer report: Reviewer response for version 1

**DOI:** 10.21956/wellcomeopenres.26438.r126118

**Published:** 2025-06-30

**Authors:** Henrique Antoniolli

**Affiliations:** 1 Universidade Federal do Rio Grande do Sul (UFRGS), Porto Alegre, Brazil

The present study reports a genome assembly for the grouse louse fly, *Ornithomya chloropus*. The Background section provides a brief overview of the species' general biology but does not clearly state the reasons for sequencing its genome. The Methods are well described, with appropriate references to protocols and software used. The assembly shows satisfactory completeness, supporting its publication.

 I approve indexing the paper but recommend that the authors include a few sentences explaining the rationale behind sequencing this genome.

Are sufficient details of methods and materials provided to allow replication by others?

Yes

Is the rationale for creating the dataset(s) clearly described?

Partly

Are the datasets clearly presented in a useable and accessible format?

Yes

Are the protocols appropriate and is the work technically sound?

Yes

Reviewer Expertise:

Genomics, evolution

I confirm that I have read this submission and believe that I have an appropriate level of expertise to confirm that it is of an acceptable scientific standard, however I have significant reservations, as outlined above.
